# Stroke and Multiorgan Failure as the Initial Manifestations of Lupus

**DOI:** 10.7759/cureus.57980

**Published:** 2024-04-10

**Authors:** Patrícia Costa, Ana Rita Silva, Adriana Carones, Sónia Teixeira, Paulo Coimbra

**Affiliations:** 1 Intensive Care Unit, Coimbra University Hospital (CHUC), Coimbra, PRT; 2 Nephrology, Coimbra University Hospital (CHUC), Coimbra, PRT; 3 Rheumatology, Coimbra University Hospital (CHUC), Coimbra, PRT

**Keywords:** neuropsychiatric manifestations, multiorgan failure, ischemic stroke, systemic lupus erythematosus, lupus nephritis, central nervous system

## Abstract

Systemic lupus erythematosus (SLE) is a persistent autoimmune disorder that manifests across a spectrum ranging from mild to severe disease, often requiring hospitalization and critical care management. We present a severe case of systemic involvement at its onset. A young woman, with a background of arterial hypertension, presented to the emergency department exhibiting a total anterior circulation stroke and exuberant symmetric lower limb edema. Her condition rapidly deteriorated with neurological impairment, respiratory failure requiring mechanical ventilation, and acute kidney injury prompting her admission to the ICU. Following clinical investigation, a diagnosis of SLE was established, according to the European League Against Rheumatism (EULAR)/American College of Rheumatology (ACR) 2019 and Systemic Lupus International Collaborating Clinics (SLICC) 2012 classification criteria. The patient underwent treatment involving high-dose corticosteroids, followed by the Euro-Lupus protocol, resulting in significant improvement, despite her severe neurological deficit at admission. Lupus is a complex disease that is often difficult to diagnose because of its potential to mimic various other conditions. Our report delves into a case of previously undiagnosed lupus leading the patient to the ICU. The clinical scenario described adds valuable insights to the understanding of lupus-related complications and their management through a multidisciplinary approach.

## Introduction

Systemic lupus erythematosus (SLE) is a multi-system autoimmune disorder of connective tissue characterized by autoantibodies, remissions, and flares, and a highly variable clinical presentation [1].

Cerebrovascular events represent one of the most common and severe neuropsychiatric manifestations and can be a direct result of SLE, manifest as a neurological symptom, or result from concurrent cardiovascular risk factors. Their prevalence vary from 3% to 20%, accounting for up to 15% of deaths in SLE but it rarely occurs as the initial manifestation of the disease. The majority of strokes related to SLE occur within one year of diagnosis, usually accompanied by disease in other organs [2,3].

Lupus nephritis (LN) is a type of glomerulonephritis that represents one of the most severe organ manifestations of the disease. Approximately 50% of patients with SLE are reported to develop LN at some point in their lifetime, with the majority experiencing it within the first five years of the disease, often as the initial manifestation [4-6].

Therefore, SLE is a persistent autoimmune disorder that manifests across a spectrum ranging from mild to severe disease, often requiring hospitalization and critical care management. The management and outcomes of these complex cases require further exploration and understanding to optimize care and improve patient outcomes in such challenging circumstances [7,8]. Despite this, the prognosis of critically ill patients with SLE who require intensive care unit (ICU) admission remains uncertain.

We present an important case of a patient who was diagnosed with SLE based on the concurrent presence of stroke and LN, resulting in her admission to the ICU.

## Case presentation

We report the case of a Caucasian non-smoker 30-year-old female who presented to the emergency department (ED) with sudden onset of altered mental status, aphasia, and right-side hemiplegia. She was found at home by a relative slumped on the floor, approximately two and a half hours after the time of last seen well. Her medical history included arterial hypertension treated with nebivolol/hydrochlorothiazide 5/12.5 mg once daily and the recent onset of edema in the lower limbs. There was no previous documentation of additional medical history, specifically concerning the musculoskeletal, mucocutaneous, and reproductive systems, or a familiar history of autoimmune disease.

Upon admission to the ED, neurological examination revealed a Glasgow Coma Scale (GCS) score of 10 (E3, V1, M6) and a National Institutes of Health Stroke Scale (NIHSS) of 30 on account of aphasia, right facial weakness, right-side hemiplegia, and conjugate eye deviation to the left. Her blood pressure was 146/115 mmHg, heart rate was 101/minute, and she was afebrile. Lung and cardiac auscultation were unremarkable. She exhibited pronounced and symmetric edema in both lower limbs, extending up to the thighs. Blood gases taken showed normal pH with compensated metabolic acidosis (bicarbonate = 16.2 mEq) by coexisting respiratory alkalosis (partial pressure of carbon dioxide = 28.0 mmHg) and hyperkalemia (5.8 mmol/L), which was promptly corrected.

Laboratory results indicated a reduced hemoglobin level (11.1 g/dL) alongside normal mean corpuscular volume (86.5 fL) and mean corpuscular hemoglobin (29.3 pg). Leucocyte and platelet counts and coagulation times were within normal range. Serum creatinine was 2.85 mg/dL (baseline creatinine of 0.6 mg/dL) with urea levels of 146 mg/dL. Urinalysis confirmed proteinuria (200 mg/dL) and hematuria. Laboratory findings are described in Table 1.

**Table 1:** Laboratory findings on emergency department admission and on stroke unit admission. ED: emergency department; MCV: mean cell volume; MCH: mean corpuscular hemoglobin; NT: not tested; LDH: lactate dehydrogenase; TSH: thyroid-stimulating hormone; ANAs: antinuclear antibodies; ANCA: antineutrophilic cytoplasmic antibody; ASMA: anti-smooth muscle antibody; BUN: blood urea nitrogen; Anti-dsDNA: anti-double-stranded DNA; Anti-SSA: anti-Sjogren's syndrome-related antigen A; Anti-SSB: anti-Sjögren's syndrome-related antigen B; Anti-RNP: anti-ribonucleoprotein; Anti-Sm: anti-Smith.

Laboratory parameters	ED admission	Stroke unit admission	Normal range
Hemoglobin (g/dL)	11.1	8.7	12.0 - 16.0
MCV (fL)	89.2	86.5	80 - 100
MCH (pg)	29.9	29.3	26 - 34
Leukocytes (x10^9/L)	6.5	8.3	3.9 - 10.2
Platelets (x 10^9/L)	245	232	150 - 400
C-reactive protein (mg/dL)	0.4	2.89	<0.5
Sedimentation rate (mm/h)	NT	93	1 - 20
LDH (U/L)	276	239	<247
Haptoglobin (mg/dL)	NT	1.77	0.3 - 2.0
Bilirubin (mg/dL)	0.3	0.2	0.2 - 1.2
Ferritin (ng/mL)	NT	715.7	30 - 300
Triglycerides (mg/dL)	NT	193	43.8 - 195.1
Albumin (g/dL)	NT	1.8	3.5 - 5.2
Urea, plasma (BUN) (mg/dL)	146	58	7.9 - 20.9
Creatinine (mg/dL)	2.85	2.39	0.55 - 1.02
Sodium (mmol/L)	134	136	136 - 146
Potassium (mmol/L)	6.1	6.5	3.5 - 5.1
Chloride (mmol/L)	106	112	101 - 109
TSH (UI/mL)	NT	1.2	0.4 - 4.0
T4 (ng/dL)	NT	0.98	0.7 - 1.5
C3 (g/dL)	NT	0.39	0.83 - 1.93
C4 (g/dL)	NT	0.1	0.15 - 0.57
ANAs (positive/negative, titer)	NT	Positive (1:1280)	<1:160
Anti-dsDNA (UI/mL)	NT	182	<7.0
Anti-SSA (positive/negative)	NT	Positive	Negative
Anti-SSB (positive/negative)	NT	Negative	Negative
Anti-RNP (positive/negative)	NT	Negative	Negative
Protein-C antibodies (positive/negative)	NT	Negative	Negative
Protein-S antibodies (positive/negative)	NT	Negative	Negative
Anti-Sm (positive/negative)	NT	Negative	Negative
Rheumatoid factor (IU/mL)	NT	<9	<20
ANCA (positive/negative)	NT	Negative	Negative
ASMA (positive/negative)	NT	Negative	Negative
Anti-beta 2-glycoprotein I (positive/negative)	NT	Negative	Negative
Anticardiolipin antibody (positive/negative)	NT	Negative	Negative
Lupus anticoagulant (positive/negative)	NT	Negative	Negative
Direct Coombs test (positive/negative)	NT	Negative	Negative
Proteinuria (mg/dL)	200	128.1	<20
Albumin/creatinine urinary ratio (mg/g)	NT	10365	<30

**Figure 1:**
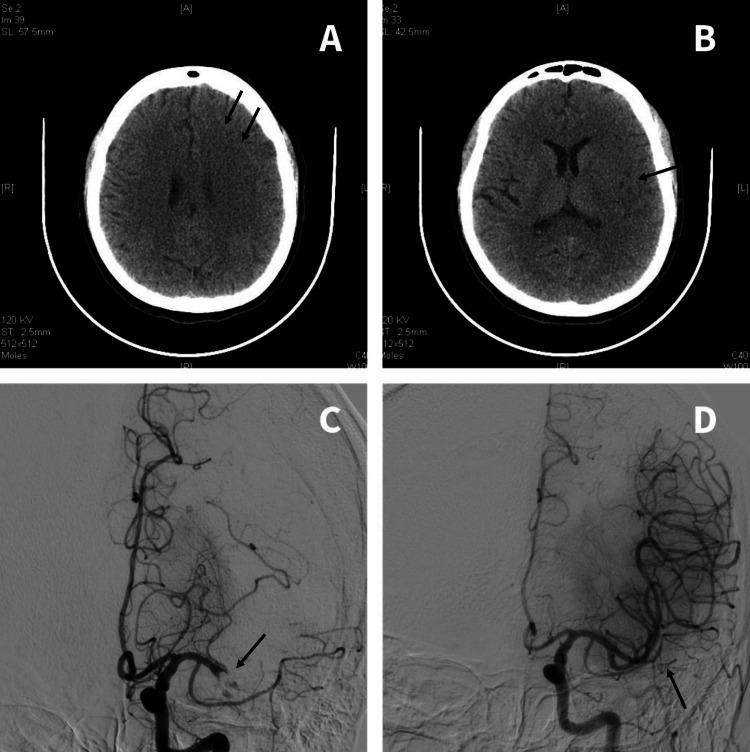
Intracranial imaging. (A) Plain head computed tomography scan revealing subtle decreased attenuation of the grey-white matter junction in the left frontal lobe (black arrows). (B) Plain head computed tomography scan revealing subtle decreased attenuation of the grey-white matter junction in the left insular regions (black arrow). (C) Computed tomography angiography revealing occlusion of the left middle cerebral artery at proximal M1 segment (black arrow). (D) Final angiographic result showing successful reperfusion (black arrow) and a final thrombolysis in cerebral infarction (TICI) score of 2b.

Head computed tomography (CT) revealed subtle decreased attenuation and swelling of the grey-white matter junction in the left frontal lobe and insular regions (Figures 1A, 1B). CT angiography showed occlusion of the left middle cerebral artery at the proximal M1 segment (Figure 1C). These features pointed toward an acute ischemic infarct in the left middle central artery (MCA) territory. She was submitted to primary thrombectomy five hours after the time of last seen well. Successful reperfusion was achieved with a final thrombolysis in cerebral infarction score of 2b (Figure 1D). Subsequently, she was admitted to the stroke unit.

Laboratory tests conducted on admission revealed an elevated erythrocyte sedimentation rate of 93 mm/h, a C-reactive protein of 2.89 mg/dL (N < 0.5 mg/dL), proteinuria in the nephrotic range (albumin/creatinine ratio of 10365 mg/g) and hypoalbuminemia (1.8 g/dL). The autoimmune panel revealed positive antinuclear antibodies (ANAs) at a titer of 1:1280, with a speckled pattern, hypocomplementemia (C3 and C4), positive anti-SSA, and a strongly positive anti-dsDNA with a titer of 182 IU/mL. On the other hand, protein-C, protein-S antibodies, anti-Sm, rheumatoid factor, antineutrophilic cytoplasmic antibody (ANCA), anti-smooth muscle antibody (ASMA), antimitochondrial antibodies (AMA), anti-RNP, anti-SSB, direct Coombs and antiphospholipid antibodies (aPL), specifically anti-beta 2-glycoprotein I, anticardiolipin antibody, and lupus anticoagulant were negative. Laboratory findings and respective evolution are shown in Table 1. Virus screening and blood cultures were also negative. Transcranial and carotid Doppler ultrasound were normal. Transthoracic echocardiography revealed a mild pericardial effusion without other notable findings.

During the initial 48 hours after being admitted to the stroke unit, the patient's condition continued to deteriorate, marked by a GCS score reduction to 9 (E3, V1, M5). Additionally, she developed acute pulmonary edema with hypoxemic respiratory failure, requiring invasive mechanical ventilation, and experienced stage III acute kidney injury, oliguria, and anasarca. A thoracic CT scan was performed, revealing bilateral large-volume pleural effusion, ground-glass opacities, and mild pericardial effusion.

She was consequently admitted to the ICU with an Acute Physiology and Chronic Health Evaluation (APACHE II) score of 23, estimating a 46% probability of death. Within the initial 24 hours of ICU admission, a neuro-imaging reassessment identified new infarct areas in the anterior cerebral artery territory and left MCA, along with parenchymal swelling. No hemorrhagic transformation occurred and 100 mg of acetylsalicylic acid per day was started.

Based on the patient’s medical history, clinical presentation, and complementary examination findings, a diagnosis of SLE was established, meeting the criteria outlined by the Systemic Lupus International Collaborating Clinics (SLICC) 2012 and the European League Against Rheumatism (EULAR)/American College of Rheumatology (ACR) 2019. Due to the patient's clinical instability, a kidney biopsy was postponed. Consequently, methylprednisolone pulses (1 g for three consequently days) were introduced immediately, followed by a maintenance dosage of 1 mg/kg/day, in addition to hydroxychloroquine 400 mg/day. Since her condition continued to deteriorate, cyclophosphamide (CYC) was promptly started according to a low-dose Euro-Lupus regimen of 500 mg IV every two weeks for a total of six doses, while corticosteroid treatment was tapered. She experienced gradual clinical and laboratory improvement, resulting in serum creatinine normalization (from 2.85 mg/dL to 0.63 mg/dL), a significant reduction in proteinuria (albumin/creatinine ratio from 10365 mg/g to 1609 mg/g), and complement levels normalization. Sequential head CT showed a significant reduction in cerebral edema and progression of ischemic areas to encephalomalacia. On the 53rd day, she was transferred to the internal medicine ward with global aphasia, ICU-acquired muscle weakness, and a GCS score of 11 (E4, V1, M6).

Although this case presented with a severe neurological deficit on admission and a prolonged ICU stay, the patient's recovery was surprisingly favorable. She was transferred to a rehabilitation facility after three months of hospitalization, where she remained for 10 weeks. At the six-month follow-up, the patient could walk unaided and perform daily activities autonomously. At this point, she was medicated with mycophenolate mofetil 2 g/day, prednisolone 5 mg/day, and hydroxychloroquine 400 mg/day. However, she still presented with Broca's aphasia, and her Glasgow Outcome Scale Extended score was 6, indicating an upper-moderate disability level.

## Discussion

Our report highlights a case of systemic involvement at the onset of SLE, characterized by rapid deterioration leading the patient to the ICU. Prior to this episode, our patient had not received a diagnosis of SLE and there were no documented manifestations that would raise suspicion of this diagnosis. However, she had recently been diagnosed with arterial hypertension and had also noticed symmetric edema in her lower limbs, which she attributed to prolonged standing during work shifts. Upon ICU admission, she satisfied the SLICC criteria by meeting five out of the 17 diagnostic criteria, including two clinical and three immunologic criteria [9]. She additionally met the EULAR/ACR 2019 criteria, satisfying four out of the 10 criteria for a definitive diagnosis of SLE, specifically serositis, proteinuria, hypocomplementemia, and a positive anti-dsDNA result [10]. It is crucial to emphasize that the SLICC or ACR/EULAR classification criteria are not diagnostic criteria and patients may manifest additional symptoms beyond those specified in these criteria, which should not be overlooked.

Assessing nervous system involvement in SLE is challenging. Reported neurological complications range from 14% to 95% in SLE cases, which can be attributed to variations in the criteria used to define SLE [11,12]. Cerebrovascular disease may be directly attributed to the disease per se, a manifestation of neuropsychiatric SLE, or the result of traditional cardiovascular risk factors [11]. The majority of strokes related to SLE occur within the first year after diagnosis; on the other hand, strokes related to atherosclerosis tend to appear in late stages [13]. Various mechanisms, such as thromboembolic disease associated with antiphospholipid syndrome, marantic endocarditis-related cardioembolism, accelerated atherosclerosis, traditional stroke risk factors, and rarely, cerebral vasculitis can contribute toward stroke development [3]. Approximately 36% of antiphospholipid syndrome patients also have SLE and the combination of these conditions can amplify the risk of cerebrovascular events [8]. Nevertheless, our patient consistently exhibited negative results for aPL in multiple immunologic studies, strongly refuting this hypothesis.

Stroke as the first manifestation of SLE is atypical. A 2018 review of the literature identified 10 similar cases, mainly in young women with previously undiagnosed SLE. The stroke mechanism was not clearly defined in these cases and treatment with immunosuppression and anticoagulation was initiated for secondary stroke prevention [13]. In our case, given the absence of aPL, the treatment approach was limited to immunosuppression, antiplatelet therapy, and cardiovascular risk factors control resulting in a favorable outcome.

It is important to timely recognize and treat kidney disease since early treatment response is related to a better prognosis. The treatment process of LN includes two stages: induction of remission and maintenance treatment. The initial induction of remission is the key stage in the treatment of severe LN patients, which generally lasts for three to six months. At this stage, glucocorticoids and immunosuppressive drugs (such as CYC, azathioprine, mycophenolate mofetil, cyclosporine, and tacrolimus) can be combined. If the condition reaches partial or complete remission, maintenance treatment can be implemented with a course of six to 24 months. Patients with complete remission can gradually reduce or terminate treatment within one year, while patients with partial remission need to continue maintenance treatment [14,15].

Despite the emergence of new biological agents, CYC is still the first-line drug widely used to treat patients with severe LN. Its combination with glucocorticoids can be more effective in preventing doubling serum creatinine levels than glucocorticoids alone [14,16]. However, in some patients, treatment has to be stopped due to infection onset, liver damage, bone marrow suppression, and leukopenia [14,16]. Considering our patient’s recurring infection episodes, the choice to persist with immunosuppression was consistently guided by an assessment of the disease's progression. Despite the delicate balance, this strategy led to positive outcomes.

Some studies indicate that CYC treatment is associated with menstrual disorders/premature ovarian failure, in relation to the cumulative dose and the patient's age at the time of treatment [17]. It is important to highlight that the decision to use CYC in this young patient was related to the severity of the situation and the current recommendations.

This case’s unique particularity was the rapid and severe form of an inaugural SLE requiring ICU admission. While SLE has emerged as the primary autoimmune disease requiring ICU admission, the predominant reasons are infections and pulmonary complications during flares [18]. The complex diagnostic workup and treatment of SLE patients with critical illness associated with flare activity or other complications of the disease is challenging in the ICU and requires close collaboration between intensivists and rheumatologists. Key mortality factors in SLE patients in the ICU include a high APACHE II score, mechanical ventilation, and vasoactive agent use. Mortality rate varies across studies, ranging from 18.4% to 78.5% [19,20].

This case emphasizes the importance of adopting a multidisciplinary approach, engaging neurologists, nephrologists, rheumatologists, neuroradiologists, intensivists, and physiatrists. Furthermore, our patient's remarkable recovery at the six-month follow-up highlights the importance of rapid diagnosis and treatment, as well as initiating early rehabilitation and maintaining it post discharge to enhance patient independence.

## Conclusions

In conclusion, this case underscores the complexity of SLE and the challenges it poses in diagnosis and management, particularly when presenting with severe multiorgan involvement as an initial manifestation. The rapid deterioration of the patient, leading to critical care admission, highlights the importance of considering lupus as a potential underlying cause in cases of acute stroke and multiorgan failure, especially in young individuals with otherwise unexplained clinical findings. This case also emphasizes the critical role of a multidisciplinary approach involving rheumatologists, neurologists, nephrologists, and intensivists in the timely diagnosis and management of SLE-related complications, particularly in the intensive care setting, where close monitoring and aggressive treatment are paramount for improving patient outcomes.

Furthermore, the favorable response to treatment, despite the severity of the initial presentation, underscores the significance of early recognition and initiation of appropriate immunosuppressive therapy in patients with lupus nephritis and neuropsychiatric manifestations. While challenges such as infection risk and treatment-related adverse effects exist, tailored treatment regimens guided by disease severity and response to therapy can lead to favorable outcomes, as demonstrated in this case. Continued research efforts aimed at elucidating the pathogenesis of SLE-related complications and refining treatment strategies are essential for further improving the prognosis and quality of life of patients with this complex autoimmune disorder.

## References

[1] Branco JC, Rodrigues AM, Gouveia N (2016). Prevalence of rheumatic and musculoskeletal diseases and their impact on health-related quality of life, physical function and mental health in Portugal: results from EpiReumaPt- a national health survey. RMD Open.

[2] Nikolopoulos D, Fanouriakis A, Boumpas DT (2019). Cerebrovascular events in systemic lupus erythematosus: diagnosis and management. Mediterr J Rheumatol.

[3] de Amorim LC, Maia FM, Rodrigues CE (2017). Stroke in systemic lupus erythematosus and antiphospholipid syndrome: risk factors, clinical manifestations, neuroimaging, and treatment. Lupus.

[4] Anders HJ, Saxena R, Zhao MH, Parodis I, Salmon JE, Mohan C (2020). Lupus nephritis. Nat Rev Dis Primers.

[5] Croca SC, Rodrigues T, Isenberg DA (2011). Assessment of a lupus nephritis cohort over a 30-year period. Rheumatology (Oxford).

[6] Imran TF, Yick F, Verma S (2016). Lupus nephritis: an update. Clin Exp Nephrol.

[7] Gasser EK, Schell-Chaple HM (2020). Systemic lupus erythematosus and critical illness. AACN Adv Crit Care.

[8] Williams FM, Chinn S, Hughes GR, Leach RM (2002). Critical illness in systemic lupus erythematosus and the antiphospholipid syndrome. Ann Rheum Dis.

[9] Petri M, Orbai AM, Alarcón GS (2012). Derivation and validation of the Systemic Lupus International Collaborating Clinics classification criteria for systemic lupus erythematosus. Arthritis Rheum.

[10] Aringer M (2019). EULAR/ACR classification criteria for SLE. Semin Arthritis Rheum.

[11] Shaban A, Leira EC (2019). Neurological complications in patients with systemic lupus erythematosus. Curr Neurol Neurosci Rep.

[12] Katkowska M, Łosoś M, Tarnacka B (2021). Systemic lupus erythematosus and critical illness polyneuropathy. Reumatologia.

[13] Ioannidis S, Mavridis M, Mitsias PD (2018). Ischemic stroke as initial manifestation of systemic lupus erythematosus: a case report and review of the literature. eNeurologicalSci.

[14] Quan XY, Chen HT, Liang SQ (2022). Revisited cyclophosphamide in the treatment of lupus nephritis. Biomed Res Int.

[15] Parikh SV, Almaani S, Brodsky S, Rovin BH (2020). Update on lupus nephritis: core curriculum 2020. Am J Kidney Dis.

[16] Bajema IM, Wilhelmus S, Alpers CE (2018). Revision of the International Society of Nephrology/Renal Pathology Society classification for lupus nephritis: clarification of definitions, and modified National Institutes of Health activity and chronicity indices. Kidney Int.

[17] Giambalvo S, Garaffoni C, Silvagni E, Furini F, Rizzo R, Govoni M, Bortoluzzi A (2022). Factors associated with fertility abnormalities in women with systemic lupus erythematosus: a systematic review and meta-analysis. Autoimmun Rev.

[18] Lee J, Dhillon N, Pope J (2013). All-cause hospitalizations in systemic lupus erythematosus from a large Canadian referral centre. Rheumatology (Oxford).

[19] Suárez-Avellaneda A, Quintana JH, Aragón CC (2020). Systemic lupus erythematosus in the intensive care unit: a systematic review. Lupus.

[20] Brünnler T, Susewind M, Hoffmann U, Rockmann F, Ehrenstein B, Fleck M (2015). Outcomes and prognostic factors in patients with rheumatologic diseases admitted to the ICU. Intern Med.

